# Perinatal Androgens and Adult Behavior Vary with Nestling Social System in Siblicidal Boobies

**DOI:** 10.1371/journal.pone.0002460

**Published:** 2008-06-18

**Authors:** Martina S. Müller, Julius F. Brennecke, Elaine T. Porter, Mary Ann Ottinger, David J. Anderson

**Affiliations:** 1 Department of Biology, Wake Forest University, Winston-Salem, North Carolina, United States of America; 2 Cold Spring Harbor Laboratory, Watson School of Biological Sciences and Howard Hughes Medical Institute, Cold Spring Harbor, New York, United States of America; 3 Animal & Avian Sciences, University of Maryland, College Park, Maryland, United States of America; University of Turku, Finland

## Abstract

**Background:**

Exposure to androgens early in development, while activating adaptive aggressive behavior, may also exert long-lasting effects on non-target components of phenotype. Here we compare these organizational effects of perinatal androgens in closely related Nazca (*Sula granti*) and blue-footed (*S. nebouxii*) boobies that differ in neonatal social system. The older of two Nazca booby hatchlings unconditionally attacks and ejects the younger from the nest within days of hatching, while blue-footed booby neonates lack lethal aggression. Both Nazca booby chicks facultatively upregulate testosterone (T) during fights, motivating the prediction that baseline androgen levels differ between obligately siblicidal and other species.

**Methodology/Principal Findings:**

We show that obligately siblicidal Nazca boobies hatch with higher circulating androgen levels than do facultatively siblicidal blue-footed boobies, providing comparative evidence of the role of androgens in sociality. Although androgens confer a short-term benefit of increased aggression to Nazca booby neonates, exposure to elevated androgen levels during this sensitive period in development can also induce long-term organizational effects on behavior or morphology. Adult Nazca boobies show evidence of organizational effects of early androgen exposure in aberrant adult behavior: they visit unattended non-familial chicks in the colony and direct mixtures of aggression, affiliative, and sexual behavior toward them. In a longitudinal analysis, we found that the most active Non-parental Adult Visitors (NAVs) were those with a history of siblicidal behavior as a neonate, suggesting that the tendency to show social interest in chicks is programmed, in part, by the high perinatal androgens associated with obligate siblicide. Data from closely related blue-footed boobies provide comparative support for this interpretation. Lacking obligate siblicide, they hatch with a corresponding low androgen level, and blue-footed booby adults show a much lower frequency of NAV behavior and a lower probability of behaving aggressively during NAV interactions. This species difference in adult social behavior appears to have roots in both pleiotropic and experiential effects of nestling social system.

**Conclusions/Significance:**

Our results indicate that Nazca boobies experience life-long consequences of androgenic preparation for an early battle to the death.

## Introduction

The diversity of vertebrate social systems has motivated many studies regarding their evolutionary origins, but few have identified the key proximate controls. The central role of the endocrine system is well recognized, functioning at the interface of the social environment and the physiology of organisms. It orchestrates behavioral responses to social cues in real time and the development of socially relevant behavioral and morphological phenotypes in ontogenic time [1]. The available data from behavioral endocrinology suggest the possibility that early exposure to androgens is required to trigger the attacks characteristic of the young of some vertebrates [2]. Studies of birds in particular have recently supported the position that nestling social interactions are mediated by circulating androgens of maternal or endogenous origin [3]. The data from developmental endrocrinology suggest that such exposure, if it occurs during developmentally sensitive periods, could induce organizational effects [4] on behavior [5], [6] and/or morphology [7]–[9] that represent pleiotropic epiphenomena. To our knowledge, these two areas of study involving hormones have not been integrated to ask the question: when androgens are mobilized in the context of sibling aggression in developing vertebrates, do those androgens also induce enduring phenotypes affecting later life stages?

Here we explore the endocrine dynamics behind two divergent neonatal social systems in closely related seabird species [10], testing predictions regarding the regulation of androgens just after hatching, when lethal aggression is expressed in one of the species, and the potential long-term consequences of this exposure. Nazca boobies (*Sula granti*) raise only one offspring at a time, as do other pelagic seabirds, but often lay a second egg that counters the poor hatching success characteristic of this species [11], [12]. Obligate siblicide solves the frequent problem of hatching both eggs: the older nestling (A-chick) unconditionally attacks its younger, smaller sibling (B-chick) and ejects it from the nest within days of hatching [13]. In facultatively siblicidal blue-footed boobies (*Sula nebouxii*), fatal sibling aggression is conditional on food availability, and, if it occurs, does so later in the nestling period [14], [15]. Nazca booby neonates thus face a high probability of lethal fighting, while blue-footed booby neonates do not.

Obligate siblicide represents the maximum challenge posed in an aggressive contest: two neonates are confined together until one kills the other. The dramatic fitness consequences of poor performance in this social system should lead to strong selection for adaptations related to siblicide in Nazca boobies, such as an endocrine milieu that facilitates the rapid onset of aggressive behavior. During fights, both Nazca booby hatchlings facultatively upregulate testosterone [T; 3], in accord with the Challenge Hypothesis [2], implicating testosterone in a quick and forceful transition to combat. While potentially critical to facilitating a rapid mobilization of T for aggression, high perinatal androgens can also induce long-term phenotypic effects, such as impaired immune function, compromised future reproduction, and developmental instabilities [8], [16]–[18]. Following the demonstration that both first- and second-hatching Nazca booby nestlings temporarily up-regulate T during fights [3], we predicted that Nazca booby neonates also maintain a higher baseline level of potentially costly androgens than do species lacking obligate siblicide. Blue-footed boobies provide the complement for a powerful comparative test of this idea, given their phylogenetic [10], morphological, behavioral, and ecological [19] similarities to Nazca boobies, including siblicidal behavior [13]. Blue-footed booby neonates receive no benefits of androgen-based aggression at hatching, so the long-term costs of early exposure to these agents should cause selection to penalize high circulating androgens related to neonatal aggression. We compare hatchling androgen levels in the two species to ask whether Nazca boobies hatch with higher concentrations of androgens than do blue-footed boobies.

Elevated steroid concentrations during developmentally sensitive periods can have organizational effects on the central nervous system and program certain facets of behavior; the effect of uterine androgen exposure on adult social phenotype of mice is a well known example [20]. Unusual social interest of non-breeding adult Nazca boobies in conspecific and heterospecific nestlings [21], [22] provides an opportunity to examine organizing effects of androgens on behavior. These Non-parental Adult Visitors (NAVs) search the breeding colony for unguarded nestlings, join them at the nest, and display parental/courtship behavior, aggression, and sexual behavior in various mixtures [21]. No satisfactory ultimate explanation for this phenomenon exists; NAV behavior confers no obvious fitness benefits on individuals [23], yet is ubiquitous among both sexes at our study site [21] and elsewhere in the species' range [Malpelo Island, Colombia, F. Estela, pers. comm]. On the proximate level, NAVs were reported to have higher corticosterone levels and lower T than non-NAVs, consistent with an activational role in this behavior [24]. We used complete life histories of birds in our long-term study population in a longitudinal test of the hypothesis that the neonatal T surges that accompany siblicide-related aggression induce organizational effects on the tendency to show NAV behavior in adulthood, by asking whether siblicidal individuals perform NAV behaviors at a higher frequency than do nonsiblicidal individuals.

While attendance of active nests by nonbreeders is a well-documented aspect of prospecting behavior in many bird species [25], this intense social interest of adults in unrelated young is virtually unreported elsewhere in the literature [21], although it may occur in other species at low frequency, until now misidentified as poor parental behavior or as anomalous. We present the first report of NAV behavior in the closely related blue-footed booby (*Sula nebouxii*) from observations performed in a dense breeding colony on Isla Lobos de Tierra, Perú. Our previous understanding of blue-footed booby behavior comes from a breeding colony in the Galápagos Islands, with inter-nest distances ranging between 3.3—377 m [26]. NAV behavior was observed on only one occasion during 46 person-years of field work in and around that colony. In Perú, however, blue-footed booby colony density was comparable to the density of the Nazca booby nests where we studied them in the colony at Punta Cevallos, Española Island, Galápagos [27], [28]. Ecological factors, such as high nesting density, may cause some populations to show NAV behavior while others do not. Here, we compare NAV behavior of the two species from similar colony environments, integrating these data into the model of behavioral organization via early androgen exposure. With the contrast in neonatal androgen level established (see Results), we compare relative frequency of NAV behavior among Nazca boobies and blue-footed boobies and examine different types of NAV behaviors separately to test the following hypothesis: if androgens associated with siblicide organize NAV aggression, then blue-footed boobies should show NAV behavior less frequently and less aggressively than do Nazca boobies.

In summary, we use both longitudinal analysis and the comparative method to examine linkage among neonatal social system, androgen concentrations, and enduring behavioral effects of early exposure to androgens. We test two hypotheses: that Nazca boobies hatch with higher baseline androgen levels than do blue-footed boobies, in parallel with the contrast in lethal fighting among hatchlings; and that participation in the different neonatal social systems induces persistent developmental consequences for behavior.

## Results

### Androgens

**Figure 1:**
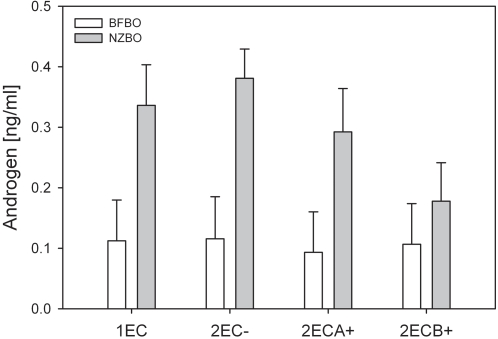
Mean (±95% CI) androgen level in Nazca booby (NZBO) and blue-footed booby (BFBO) hatchlings. 1EC = products of one-egg clutches, 2EC- = products of two-egg clutch where only one egg hatched, 2ECA+ = first hatchling from two-egg clutch where both eggs hatch, 2ECB+ = second hatchling from two-egg clutches where both eggs hatch.

All nestlings providing androgen (combined 5α-DHT and T) samples came from one- or two-egg clutches, and were categorized into four types: product of single-egg clutch, product of a two-egg clutch in which only one egg hatched, first nestling in a two-nestling brood, and second nestling in a two-nestling brood. ANOVA of androgen level, incorporating species, nestling type, and sex effects, and their interactions, revealed a significant species effect (Table 1, Fig. 1). Nestling history and some interaction terms also explained significant variation in androgen level (Table 1), but the species difference was the largest component of variance by far, with an ANOVA mean square exceeding that of the second largest component (nestling history) by a factor of 14 (Table 1). Removal of the non-significant effects from the model simplified the result, rendering the species * nestling history * sex interaction non-significant (F_3,131_ = 2.147, P = 0.10), and maintaining the strong species effect (F,_131_ = 78.814, P<10^−6^) and lesser nestling history (F_3,131_ = 6.702, P = 0.0003) and nestling history * sex effects (F_1,131_ = 2.954, P = 0.035). With the exception of second-hatching nestlings, which typically are killed shortly after hatching, Nazca booby neonates had three times or more the androgen level of comparable blue-footed boobies (Fig. 1).

**Table 1:** ANOVA results comparing androgen levels of Nazca and blue-footed boobies, incorporating effects of nestling type and sex and their interactions.

Effect	d.f	MS	F	p
Intercept	1	488.106	1412.126	<10^−6^
Species	1	27.884	80.672	<10^−6^
Nestling history	3	1.925	5.570	0.001
Sex	1	1.198	3.466	0.065
Species * Nestling history	3	0.748	2.163	0.096
Species * Sex	1	0.441	1.275	0.261
Nestling history * Sex	3	1.289	3.728	0.013
Species*Nestling history * Sex	3	0.981	2.839	0.041
Error	126	0.346		

### Behavior

**Figure 2:**
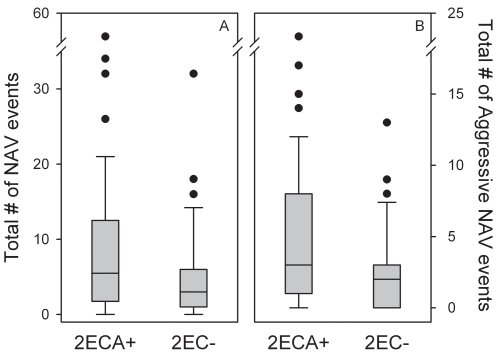
Number of NAV events (A: total, B: aggressive) performed by adults derived from two-egg clutches. Siblicidal individuals (2ECA+) are contrasted with non-siblicidal individuals (2EC-) whose sibling egg failed to hatch. Boxes and whiskers represent the middle 50% and middle 80% of the data, respectively.

Longitudinal data from Nazca boobies derived from two-egg clutches showed that siblicidal nestlings mature into adults that exhibit more NAV behavior than do non-siblicidal conspecifics (t-value = 2.468, df = 104, N = 108, *P* = 0.015; Fig. 2A). Specifically, siblicidal Nazca boobies displayed more frequent aggressive NAV behavior as adults (t-value = 2.287, df = 104, N = 108, *P* = 0.024; Fig. 2B), whereas the frequencies of affiliative and sexual NAV behavior did not vary significantly with nestling social experience (t-value = 1.304, df = 104, N = 108, *P* = 0.195; t-value = 0.006, df = 104, N = 108, *P* = 0.995).

We detected a far lower frequency of NAV behavior in Peruvian blue-footed boobies. When expressed as the number of NAV events per non-breeder present, scaled to observation effort, Nazca and blue-footed boobies performed 0.0725 (95% CI = 0.0588–0.0862) and 0.0017 (95% CI = 0.0014–0.0018) NAV events/nonbreeder/hour, respectively. Scaled to the number of unattended chicks available, we observed 0.0586 (95% CI = 0.0.0489–0.0683) and 0.0152 (95% CI = 0.0138–0.0166) NAV events/unattended chick/hour in Nazca boobies and blue-footed boobies, respectively. In each of these comparisons, neither mean is included in the other mean's 95% CI. We predicted that if early androgen exposure organizes aggressive NAV behavior in boobies, then aggressive NAV interactions should represent a smaller fraction of the NAV repertoire in blue-footed boobies than in Nazca boobies. We found support for this hypothesis: in most years, Nazca booby NAV events included significantly more aggressive and less affiliative behaviors compared to blue-footed booby events (Table 2; log-linear analysis, Nazca booby frequencies differ from those of blue-footed boobies [P<<α_crit_] in all years except 2001–02 [P = 0.24]).

**Table 2:** Percentage of behavior types observed in NAV events, by species.

Nazca boobies				
Year	Affiliative	Aggressive	Sexual	N
2000–01	34.4	49.5	16.0	2169
2001–02	45.0	41.9	13.1	1263
2002–03	34.1	57.5	8.4	2069
2004–05	37.1	56.7	6.3	2638

Blue-footed boobies				
Year	Affiliative	Aggressive	Sexual	N
2006–07	50.3	35.2	14.5	165

Most Nazca booby chicks show physical evidence of past aggressive NAV attacks, including lacerations and bare patches of skin where down has been scraped away [21]. In contrast, we found 0 out of 1473 medium-sized blue-footed booby chicks had abrasions or missing down, suggesting that while aggressive NAV interactions occur in blue-footed boobies, they are a milder form of the phenomenon. The absence of such markers is apparently not attributable to higher durability of blue-footed boobies' plumage or skin, because Nazca booby NAVs visit blue-footed booby chicks at our Galápagos study site and cause visible injuries to them [22].

## Discussion

We found that hatchling Nazca boobies, facing a strong possibility of an imminent fight to the death, emerge from the egg with a higher circulating androgen level than do blue-footed boobies, providing comparative evidence of the role of androgens in sociality. With the exception of B-chicks, which typically are killed shortly after hatching, Nazca booby neonates had three times or more the androgen level of comparable blue-footed boobies. Among these Nazca booby hatchlings, androgen level was not sensitive to the post-natal stimuli of the presence of a potential competitor (an egg) or to position in the laying sequence (Fig. 1), suggesting that levels on the day of hatching reflect a species difference in exposure to androgens extending back in time into the embryonic period, rather than facultative upregulation after hatching.

Within the Nazca booby population, more than half of the clutches contain two eggs in most years [29], [30], and 68% of two-egg clutches produce two hatchlings [11], so more than 1/3 of all Nazca boobies experience the additional early androgen exposure associated with upregulation of T during obligate siblicide [3]. Given the evidence that neonatal birds with altricial development experience organizational effects from post-hatching exposure (the first 30% of the nestling period) to steroid hormones [31], the early exposure of highly altricial Nazca boobies to a high androgen level is expected to coincide with a sensitive period in development. Accordingly, we found evidence of organizational effects of siblicide-related androgens in aberrant adult behavior. Siblicidal Nazca boobies showed more frequent social attraction (NAV behavior) to unguarded chicks years later as a nonbreeding adult, compared to non-siblicidal Nazca boobies (Fig. 2A). This predictive ability of siblicidal history was due to a significant association with the frequency of aggressive NAV interactions (Fig. 2B); it did not predict the frequencies of the affiliative or sexual components of NAV behavior. These results provide rare evidence from a natural population that social behavior (in this case, aggressive interest in non-familial young) expressed as an adult is conditioned by androgen exposure while young, which itself is related to the extreme social challenge faced by neonatal Nazca boobies.

Nazca boobies provide a rare example of a non-human animal known to express such widespread and varied social interest in unrelated young. Adolescent male, but not female, yellow-rumped caciques (*Cacicus cela*) persistently attack and attempt to copulate with fledglings, with unknown fitness consequences [32]. Black-legged kittiwake (*Rissa tridactyla*), nonbreeders also visit active nests where parents are absent, although this “squatting” behavior is interpreted as an assessment of site quality, important for territory acquisition [33]. Neither of these cases nor more anecdotal reports seem to represent parallels to the Nazca booby situation, in which the motivation to visit active nests is clearly social interaction with a chick, with fully mature adults of both sexes engaging in a variety of social behaviors. The aggressive component of NAV behavior of Nazca boobies shows an ontogenic linkage with androgen exposure experienced during siblicide events. This result prompts the expectation that the elevated androgens of Nazca boobies before siblicide, compared to blue-footed boobies (Fig. 1) and probably most other birds, also lead to the later expression of NAV behavior. Consistent with this expectation, NAV behavior occurs at low frequency in Peruvian (this study) and Galápagos (personal observation) blue-footed booby populations, and is unreported from central American colonies. Moreover, aggression represents a lower proportion of the NAV repertoire of blue-footed boobies compared to Nazca boobies (Table 1). The contrast in the NAV phenomenon among the two species is consistent with the hypothesis that perinatal androgens organize propensity to show NAV behavior, in a behavioral cascade rooted in the hatchling social environment. Experimental manipulation of perinatal androgen exposure in these species, with longitudinal followup of adult social behavior, can evaluate this idea further.

Launching their infants into vastly different social systems, Nazca and blue-footed boobies otherwise exhibit similar ecologies, life histories, and phylogenies, minimizing potentially confounding variables that would interfere with identification of the proximate regulators of these social systems. These species also provide an opportunity to assess life-long consequences of neonatal social system dynamics on phenotype via these proximate mechanisms. Here we demonstrate links between neonatal social system, androgen concentrations, and persistent phenotypic effects of early exposure to androgens. We suggest that the strong selection for the androgens that facilitate obligately siblicidal behavior in Nazca boobies outweighs any pleiotropic consequences arising later in life.

## Materials and Methods

### Blood sampling and assays

As part of a long-term study on this species we monitored over 16,000 Nazca booby nests at Punta Cevallos, Isla Española, (89° 37′ W, 1° 23′ S) in the Galápagos Islands since 1984 in which laying dates, laying order, clutch size, hatching dates, and hatching order were recorded. We sampled first (CA) and second (CB) chicks from 15 nests with two egg clutches where both eggs hatched (2ECA+ and 2ECB+, respectively), 15 nests with two egg clutches where either the first egg or the second egg was present at hatching of the other egg but did not hatch itself (2EC-), and 15 chicks from one-egg clutches (1EC). Chicks were sampled within 24 hours of hatching, usually between 10am–1pm. Approximately 200 µl of blood was taken from the brachial vein using a 27 ½ gauge needle and unheparinized microcapillary tubes and then collected in 1.5 mL Eppendorf microcentrifuge tubes. Typically, sampling time was 3 min., in rare cases 5–8 min. Samples were processed within 1–2.5 hrs of collection. We centrifuged blood samples in the field for 10 minutes, then removed a known volume of serum which was stored separately in tubes containing 750 µl absolute EtOH. Red blood cells were resuspended in ca. 500 µl of 70% EtOH. All samples were stored at ambient temperature for 1 month before refrigeration upon arrival at Wake Forest University. Hormones were extracted and assayed at the University of Maryland. DNA was extracted from red blood cells for PCR sex identification at Wake Forest University [34].

We monitored 925 blue-footed booby nests in Dec 2006 on Isla Lobos de Tierra (80° 51′ W, 6° 24′ S), Perú which already contained eggs, using only nests that contained one- or two-egg clutches. We collected blood samples from chicks within 24 hours of hatching using the same procedure used for Nazca boobies. We sampled 16 first and second hatchlings from two-egg clutches (2ECA+ and 2ECB+, respectively), 16 chicks from two-egg clutches where one egg did not hatch (2EC-), and 16 chicks from one-egg clutches (1EC). Hormone samples were extracted at Wake Forest University and assayed at the University of Maryland, DNA was extracted from red blood cells for PCR at Wake Forest University.

After double ether steroid extraction (85% recovery), androgens (5α-DHT and T) were assayed via radioimmunassay (RIA; 35) for chicks from both species. The RIA was validated for parallelism, sensitivity (10pg/ml), accuracy, and precision (<10% CV) for serum from both species.

### Behavioral observations

#### Nazca Boobies

In January 2005, we placed distinctive blue plastic bands on all adult nonbreeders and failed breeders that had known nestling histories (age, clutch size, hatching order and siblicidal/non-siblicidal) in the “study area”, a subsection of the Nazca booby colony at Punta Cevallos [28]. The numbers on the bands could be read easily from a distance. From January 19, 2005–Mar 31, 2005, each afternoon between ca. 1300–1630 hrs, two observers systematically patrolled the study area recording all NAV events, the identity of the NAVs, and the nest number of the chick victims (504 person/hrs in total). Any NAV interaction of the three classes between a plastic-banded nonbreeder and a chick was recorded as a NAV event; repeated NAV interactions of a given behavior type between the same individuals in one day were all considered as the same “NAV event” [23]. NAV behavior classes included the following: aggressive (biting, shaking, or jabbing), affiliative (attending the chick with little interaction, preening, presenting gifts of feathers or pebbles), or sexual (attempted copulation with chick). Some analyses used an extended data set to include NAV observations recorded during the breeding seasons of 2001–02 and 2002–03 using the same methods.

In 2004–05, an average of 33.0 banded Nazca booby nonbreeders (SD = 17.0) was present at mid-day in the observation area over the course of the NAV observation period. During the subsequent afternoon hours, we observed an average of 9.22 active NAVs (SD = 5.1) in the study area and a mean of 1.85 NAV events per person-hour (SD = 1.28; n = 1750 NAV events). The study area had an average of 130.3 medium-sized chicks in the colony (>than 20 days old; <fledging age), 66.8 of which (SD = 19.2) were unattended on average.

Correct assessment of the relative frequency of NAV behavior required adjustment for variation in colony attendance among individuals. We performed nightly band re-sight surveys of the study area in which we noted all plastic banded birds. Only birds present on at least 5 nights during the period of the NAV observations were considered colony residents and all others were excluded from the analysis. In addition, a bird had to be of “nonbreeder” status on at least one of the nights present to be included in the analysis as a potential NAV.

#### Blue-footed Boobies

We conducted systematic behavioral observations in a large blue-footed booby colony on Isla Lobos de Tierra, Perú (80° 51′ W, 6° 24′ S) in Dec 2006. Patrolling a subsection of the colony, we recorded all NAV behaviors and categorized them as aggressive, affiliative, or sexual, and noted the time of day. We performed 30 hours of behavioral observations over the course of five days (29 Dec 2006 to 2 Jan 2007; Day 1: 1000–1600 hrs; Days 2–4: 930–1630 hrs; Day 5: 930–1230 hrs). We patrolled an area with hundreds of unattended medium-sized chicks (between 20–100 days old), and even higher numbers of non-breeding adults, in which abundant opportunity for NAV interactions existed. Because adults were unbanded and so unrecognizable, we were unable to determine how many, if any, adults were involved in repeated NAV events with a particular chick. As a consequence, our assumption that each NAV event was independent may not be strictly true. To perform a single loop of the area required 30 minutes; for each pass of the colony we recorded each NAV interaction with a given chick as a new event due to the ample number of nonbreeders in the colony and relative high probability that the NAV was a new bird. We distinguished affiliative NAVs from parents by observing the chicks' responses to the attending adults, as in Nazca booby chicks [21]: blue-footed booby chick victims of NAV behavior tuck their bills under and push their forehead to the ground, leaving the backs of their necks exposed in a submissive posture, during the interactions.

An average of 1950.7 nonbreeders (SD = 90.1) were present during the middle of the day in the observation area during the five days of behavioral observations, and we observed a mean of 3.23 NAV events per person-hour (SD = 0.33, n = 165 NAV events). Chick attendance by blue-footed booby parents (85.6%) was higher than by Nazca boobies (44.4%), which may have limited opportunity for blue-footed booby nonbreeders to show NAV behavior. However medium-sized chicks (>20 days, <fledging age) were abundant: the observation area had 1473 medium-sized chicks and an average of 212.3 (SD = 31) chicks that were unattended, or three times the number of available Nazca boobies at Punta Cevallos.

### Statistical Analyses

We compared androgen levels using a three-way ANOVA (main effects species, sex, and nestling type, after checking homogeneity of variances using Levene's Test (F_15, 126_ = 1.73, P>0.05) and normality using a normal probability plot of residuals. Untransformed data showed a tendency to non-normality, which was corrected by log-transformation, and we used log-transformed androgen level in the ANOVA.

To test the organizational effects hypothesis with data from Nazca boobies, using R [38], we fitted a generalized linear mixed model to frequency of total NAV events of different types performed by an individual, using nestling history, sex, and age as fixed effects and number of nights presents in the colony as a nonbreeder as a random effect. Nestling history was a dichotomous variable describing whether or not a nonbreeder derived from a two-egg clutch was siblicidal as a chick. The residuals of the dependent variables followed a negative binomial, or “zero-inflated Poisson” distribution, so we specified the model for an “overdispersed Poisson” distribution. Using the t-value output, we determined significance with a two-tailed test with α = 0.05.

To compare the frequencies of NAV behavior types across species, we first used a log-linear analysis on Nazca booby data alone to test for temporal variation across years, and found a significant year x behavior type interaction (Maximum Likelihood χ^2^ = 202.05, df = 6, P<0.01). As a result, we did not collapse all Nazca booby data into a single sample for comparison with the single year of data from blue-footed boobies, instead comparing each year of Nazca booby data with the single year of blue-footed booby, adjusting the P values for multiple comparisons with the false discovery method [36], [37]. The false discovery method computes a critical α level (α_crit_) for each comparison, to which the P value is compared to determine significance.
